# Dual-mTOR Inhibitor Rapalink-1 Reduces Prostate Cancer Patient-Derived Xenograft Growth and Alters Tumor Heterogeneity

**DOI:** 10.3389/fonc.2020.01012

**Published:** 2020-06-23

**Authors:** Federico La Manna, Marta De Menna, Nikhil Patel, Sofia Karkampouna, Maria Rosaria De Filippo, Irena Klima, Peter Kloen, Lijkele Beimers, George N. Thalmann, Rob C. M. Pelger, Estela Jacinto, Marianna Kruithof-de Julio

**Affiliations:** ^1^Department for BioMedical Research, Urology Research Laboratory, University of Bern, Bern, Switzerland; ^2^Department of Urology, Leiden University Medical Center, Leiden, Netherlands; ^3^Department of Biochemistry and Molecular Biology, Robert Wood Johnson Medical School, Rutgers, The State University of New Jersey, Piscataway, NJ, United States; ^4^Institute of Pathology and Medical Genetics, University Hospital Basel, University of Basel, Basel, Switzerland; ^5^Department of Orthopedic Trauma Surgery, Academic Medical Center, Amsterdam, Netherlands; ^6^Department of Orthopedic Surgery, MC Slotervaart, Amsterdam, Netherlands; ^7^Department of Urology, Inselspital, Bern University Hospital, Bern, Switzerland

**Keywords:** bone metastasis, PDX, mTOR, disulfiram, prostate cancer, ALDH

## Abstract

Bone metastasis is the leading cause of prostate cancer (PCa) mortality, frequently marking the progression to castration-resistant PCa. Dysregulation of the androgen receptor pathway is a common feature of castration-resistant PCa, frequently appearing in association with mTOR pathway deregulations. Advanced PCa is also characterized by increased tumor heterogeneity and cancer stem cell (CSC) frequency. CSC-targeted therapy is currently being explored in advanced PCa, with the aim of reducing cancer clonal divergence and preventing disease progression. In this study, we compared the molecular pathways enriched in a set of bone metastasis from breast and prostate cancer from snap-frozen tissue. To further model PCa drug resistance mechanisms, we used two patient-derived xenografts (PDX) models of bone-metastatic PCa, BM18, and LAPC9. We developed *in vitro* organoids assay and *ex vivo* tumor slice drug assays to investigate the effects of mTOR- and CSC-targeting compounds. We found that both PDXs could be effectively targeted by treatment with the bivalent mTORC1/2 inhibitor Rapalink-1. Exposure of LAPC9 to Rapalink-1 but not to the CSC-targeting drug disulfiram blocked mTORC1/2 signaling, diminished expression of metabolic enzymes involved in glutamine and lipid metabolism and reduced the fraction of CD44^+^ and ALDEFluor^high^ cells, *in vitro*. Mice treated with Rapalink-1 showed a significantly delayed tumor growth compared to control and cells recovered from the tumors of treated animals showed a marked decrease of CD44 expression. Taken together these results highlight the increased dependence of advanced PCa on the mTOR pathway, supporting the development of a targeted approach for advanced, bone metastatic PCa.

## Introduction

Development of bone metastasis involves 65–75% of breast and prostate cancer patients with advanced, metastatic disease (1) with the axial skeleton as the most common site of bone metastasis (2).

The clinical implications revolving around the development of a bone metastatic disease include the development of skeletal-related events, like pathological fractures, spine chord compression or bone pain and represent a common event in advanced breast and prostate patients, greatly affecting their quality of life (1, 3). Bone metastasis are frequently characterized by a long latency period, characterized by the presence of subclinical micrometastasis in the bone that are difficult to detect and to target. Once symptomatic, bone metastasis is frequently associated with a progressed, highly malignant relapse of the disease (3). Despite its relevance, the study of bone metastasis has been hindered by the difficulty of obtaining high quality specimens from bone lesions (4, 5).

The dependence of prostate tissue on androgen receptor (AR) signaling prompted the development of AR-targeting molecules, like abiraterone and enzalutamide, for the treatment of metastatic castration-resistant PCa (mCRPC). However, prolonged treatment with these types of drugs fosters the molecular evolution of PCa, increasing its propensity to metastasis formation and to overcome castration (6). Therefore, new and alternative approaches are currently being investigated to overcome or limit this clinically relevant behavior. Patient-derived xenografts (PDX) have proven to be highly valuable tools for the development of precision medicine strategies for the study of PCa (7). BM18 and LAPC9 are bone metastatic PCa models with different molecular and histological features, with androgen-dependent and -independent growth, respectively (8, 9). A relevant advantage of using PDX models is the possibility of investigating cancer stem cells (CSC), a widely recognized hypothesis that accounts for the establishment of a low-cycling, drug-resistant subpopulation of cells with tumor re-growth potential (10–12).

In prostate cancer, CSC have been identified by different parameters, including surface marker expression, subpopulation-specific stainings and functional assays, with various degrees of overlap between the different methods (13, 14).

The link between aldehyde dehydrogenase (ALDH) activity, cell stemness and self-renewal potential, initially found to detect leukemia tumor-initiating cells, was then confirmed also in PCa where it associates with a potentially clinically relevant subpopulation of cells (15–17).

Pharmacological approaches to target the CSC subpopulation of PCa are currently being explored and include disulfiram, a drug used for the treatment of alcohol abuse and currently investigated for its activity against CSC in various tumors, including prostate cancer, glioblastoma and melanoma (11, 18). Multiple mechanisms of the anti-CSC action of disulfiram have been elucidated and include its primary action in the irreversible inhibition of aldehyde dehydrogenase (ALDH), inhibition of ubiquitin-E3 ligase activity, inhibition of epithelial-to-mesenchymal transition (EMT) and increase of reactive oxygen species (ROS) (12, 18). The latter two mechanisms are dependent on the availability of copper as a co-factor, forming equimolar chelation complexes with disulfiram.

The mammalian target of rapamycin (mTOR) is an atypical protein kinase that can participate in two distinct signal transduction complexes, mTORC1 and mTORC2, regulating a plethora of key cellular functions like cell growth, proliferation, survival and metabolism (19, 20). mTORC1 and 2 integrate nutrient availability status with the anabolic needs of the cell. Deregulation of the PI3K/Akt/mTOR pathway in cancer has been well-established and different clinical studies have found an overactivation of this pathway in ~40% of breast cancers and 50% of primary prostate cancers (21–23). Targeting the AR pathway with androgen blockers increases the activation of the PI3K/Akt/mTOR pathway (24). Conversely, PTEN exerts a regulatory role on the AR, acting both as AR inducer, via an Egr1- and c-Jun-mediated mechanism, and as an AR repressor, by controlling the negative AR regulator Nkx3.1 (25, 26). Recently, AR- and mTOR signaling-dependent metabolic rewiring of PCa cells and during CRPC progression was shown (27). Phase I/II trials on PCa using rapamycin analogs (rapalogs), which inhibit only a subset of mTORC1 functions, revealed clinical inefficacy (28). ATP-competitive mTOR inhibitors, which block both mTORC1 and mTORC2 kinase activity, and dual PI3K/mTOR inhibitors also showed poor efficacy in the clinic due to toxicity (29, 30). Rapalink-1, a bivalent compound that combines the durable effect of rapamycin and dual mTORC1/mTORC2 inhibition, has been developed recently (31). It remains to be examined whether Rapalink-1 would be efficacious for PCa therapy.

The aim of the present study was to determine the impact of the third generation mTOR-inhibiting compound Rapalink-1 using bone-metastatic PCa PDX models *in vitro, ex vivo*, and *in vivo*. We investigated the effects of Rapalink-1 treatment on the CSC compartment and further compared its effects to the CSC-targeting compound disulfiram, exploring the effects of mTOR blockade on the CSC subpopulation.

## Materials and Methods

### Patient Samples

Samples were collected from patients undergoing orthopedic surgery for bone metastasis (prostate cancer, 5 patients; breast cancer, 4 patients) and anonymously analyzed according to the Dutch Medical Research Involving Human Subjects (WMO) act. Samples were either immediately snap-frozen at the time of surgery for further molecular analyses or shipped in Dulbecco modified essential medium (DMEM) supplemented with 1% penicillin/streptomycin (pen/strep) and 1% Glutamax (Thermo Fisher Scientific) for organoids generation.

### RNA Isolation and RNA Sequencing

Five mm by 5-mm snap-frozen bone metastasis samples were placed in a tube with 1 ml Tripure reagent (Sigma-Aldrich) and a metallic bead and homogenized with the TissueLyser II (Qiagen) for 2 cycles of 3 min at 30 Hz. In between cycles, samples were incubated at −20°C for 5 min. Manufacturer's protocol was then followed to extract RNA from the homogenized samples. RNA quality was assessed by Bioanalyzer 2100 (Agilent Technologies) using the Nano kit and following manufacturers protocol. Samples with an “RNA Integrity Number” (RIN) > 7 were further processed for RNA sequencing. Specimens were prepared for RNA sequencing using the “NEBNext Ultra II Directional RNA Library Prep Kit for Illumina” (NEB #E7760S/L) as described previously (32). Briefly, mRNA was isolated from total RNA using the oligo-dT magnetic beads. After fragmentation of the mRNA, a cDNA synthesis was performed. This was used for ligation with the sequencing adapters and PCR amplification of the resulting product. The quality and yield after sample preparation was measured with the Fragment Analyzer. The size of the resulting products was consistent with the expected size distribution (a broad peak between 300 and 500 bp). Clustering and DNA sequencing using the NovaSeq6000 was performed according to manufacturer's protocols. A concentration of 1.1 nM of DNA was used. Image analysis, base calling, and quality check was performed with the Illumina data analysis pipeline RTA3.4.4 and Bcl2fastq v2.20. Sequence reads were aligned using STAR two-pass to the human reference genome GRCh37 (33). RSEM was used to obtain FPKM (fragments per kilobase of exon model per million reads mapped) counts. We removed duplicated gene names when present, keeping the one with highest expression. Gene counts were quantified using the “GeneCounts” option in STAR. Per-gene counts-per-million (CPM) were computed and log2-transformed adding a pseudo-count of 1 to avoid transforming 0. Genes with log2-CPM <1 in more than two samples were removed. Principle component analysis was performed using the top 200 most variable genes. Differential expression analysis was performed using the edgeR package (34). Normalization was performed using the “TMM” (weighted trimmed mean) method and differential expression was assessed using the quasi-likelihood F-test. Genes with FDR <0.05 and >2-fold were considered significantly differentially expressed.

### Immunohistochemistry and Histological Stainings

Four-μm thick sections of FFPE blocks were cut, stained for haematoxylin and eosin and mounted with Entellan (Merck-Millipore). For Ki67 and panCK stainings, cut sections were processed for antigen retrieval by pressure cooker for 10 min in citrate buffer at pH 6.0. Sections were allowed to cool and then extensively washed in running water. Endogenous peroxidases were blocked by incubation with 3% H_2_O_2_ for 15 min at room temperature. Sections were then washed twice with PBS and blocked with a solution of 3% BSA in PBS-Tween 20 0.1% (PBS-T) for 1 h at room temperature then incubated overnight with 100 μl of anti-Ki67 (1:400, rabbit), anti-panCK (1:100, mouse), rabbit IgG or mouse IgG as appropriate, see Supplementary Information for a list of the used antibodies. Sections were then washed once with PBS-T and twice with PBS before incubation for 30 min with 100 μl of EnVision anti-rabbit or anti-mouse (Agilent Technologies). Sections were then washed once with PBS-T and twice with PBS and developed in a freshly prepared AEC solution (Dako) until sufficiently developed. Sections were then washed in H_2_O and counterstained with hematoxylin before mounting with Entellan. Slides were digitalized with the Pannoramic 250 Flash III slide scanner (3D Histech).

### Western Blot

Cells were lysed in RIPA buffer (50 mM Tris-HCl pH 8.0, 100 mM NaCl, 5 mM EDTA, 0.2% SDS, 0.5% sodium deoxycholate, 1% Triton-X100) supplemented with protease and phosphatase inhibitors (cOmplete Mini, protease inhibitor cocktail and PhosStop, both by Merck Millipore). Tissue pieces were homogenized with TissueLyser II (Qiagen) for 1 cycle of 2 min at 20 Hz in RIPA buffer, using a metallic bead. Organoids were resuspended in 150 μl of RIPA buffer and homogenized with a 0.3 ml syringe. Homogenized samples were centrifuged for 15 min at >16,000 g at 4°C and supernatant collected. Protein concentrations were measured by Bradford assay and about 10–30 μg of samples were used for SDS-PAGE. Proteins were transferred onto Immobilon-PVDF (Millipore). Blots were incubated with primary antibodies overnight followed by washing with PBS-Tween, see Supplementary Information for a list of the used antibodies. Blots were then incubated with either anti-mouse or –rabbit secondary antibody. After washing with PBS-Tween, images were visualized using Supersignal ECL detection kit (ThermoFisher) and captured using Amersham Imager 600 (GE).

### Animals Maintenance and *in vivo* Experiment

Animal experiments were conducted according to Bern cantonal guidelines. Mice had unrestricted access to food and fresh water and housed in max 5 animals per cage. For xenograft surgery, nine 5-week old male CB17/SCID mice were anesthetized by subcutaneous injection with a cocktail of medetomidin (Dorbene) 1 mg/kg, midazolam (Dormicum) 10 mg/kg, and fentanyl 0.1 mg/kg. Under sterile hood, two 3 mm long incisions were performed on each side in the scapular region and a small pocket was created by lifting the skin with forceps. Freshly harvested 2 mm^3^ tumor pieces were inserted into the pockets, that were closed with resorbable 6-0 suture (Vicryl 6-0, Ethicon). Anesthesia was reversed by subcutaneous injection with atipamezol (Revertor®) 2.5 mg/kg and flumazenil (Anexate®) 0.5 mg/kg, together with buprenorphine (Temgesic) 0.1 mg/kg for analgesia, and sutured wound was disinfected with a iodopovidone solution. Three days post-implantation animals were divided into 2 groups, stratified by weight. Group 1 received 3.5 μl/g of vehicle (20% DMSO, 40% PEG-300 and 40% PBS) i.p. once a week while group 2 received Rapalink-1 (1.5 mg/Kg) resuspended in vehicle, i.p. every 5–7 days. Mouse weight, tumor size and signs of acute toxicities were monitored twice a week, tumor size was tracked by palpation and referred to standardized size beads, to minimize animals' discomfort during the experiment. Mice were euthanized as soon as signs of acute toxicity were detected or when tumor size reached 8 mm.

### Organoid Culture

Tissues were collected in basis medium [Advanced D-MEM/F-12 (ThermoFisher Scientific) supplemented with 1 ml Primocin (Invivogen), 1% GlutaMAX and HEPES 10 mM (ThermoFisher Scientific)], finely minced with a scalpel and incubated in 5 mg/ml collagenase type II (Gibco), supplemented with 15 μg/ml DNase I (Sigma-Aldrich) and 10 mM Y-27632, at 37°C for 1–3 h with occasional mixing, until completely digested. Cell suspension was then centrifuged at 400 rcf for 5 min and washed with basis medium. Cell pellet was then incubated at 37°C in 2 ml TripLE Express (ThermoFisher Scientific) for 10 min, pipetting cell suspension every 5 min. Digested cell suspension was passed through a 50 μm-pore size strainer (Celltrics, Sysmex) and washed with basis medium. When required, cells were incubated for 5 min in erythrocytes-lysing buffer to eliminate red blood cells, then washed with basis medium. Cells were counted with trypan blue with an automated cell counter (TC20, Bio-Rad), centrifuged and resuspended in complete prostate cancer organoid medium [see Supplementary Information for the complete recipe, reproduced from (35)] at 300,000 cells/ml and seeded in 1.5 ml volume in 6-well ultra-low attachment plates (ULA plates, Corning). Fresh medium was added every 2–3 days until organoids were used for downstream applications. For drug pre-treatment, LAPC9 and BM18 organoids were cultured in 6-well ULA plates in complete PCa medium for 48 h, then medium was replaced with fresh medium containing the target drug at the reported concentration and organoids were cultured for further 48 h before proceeding with downstream analysis.

### Drug Assay

Organoids were collected in basis medium and centrifuged for 3 min at 100 rcf, then they were resuspended in TripLE Express and incubated at 37°C with occasional resuspension until completely dissociated. Cell suspension was then washed with basis medium and centrifuged at 300 rcf for 5 min. Cells were resuspended at 175,000 cells/ml in complete PCa organoids medium and seeded in 20 μl volume in a 384-well low-attachment plate, with black walls (Corning). After 48 h, wells with organoids were treated with the appropriate compound, resuspended in 20 μl of PCa organoids medium. The compounds used were Rapalink-1 (10–0.001 μM, ApexBio), rapamycin (10–0.1 μM), everolimus (1–0.1 μM), abiraterone (1 μM, in EtOH), enzalutamide (10 μM), disulfiram (10–0.1 μM, Sigma-Aldrich), DMSO (0.1%), and EtOH (0.1%). Where not stated otherwise, the compounds were from Selleckchem, and were resuspended in DMSO. Wells treated with disulfiram were additionally supplemented with copper-gluconate 1 μM (Sigma-Aldrich). Each condition was assessed in quadruplicates, each experiment was repeated 3 times for the PDX models and 2 times for the patient-derived bone metastasis material.

### *Ex vivo* Tissue Culture

Freshly collected LAPC9 and BM18 tissues were aseptically cut into 1 mm-thick serial slices. Tissue slices were then carefully placed on the membrane of a 0.4 μM pores polypropylene 24-well transwell (ThinCert, Grainer Bio-one International) and cultured on 0.5 ml of DMEM supplemented with 10% FBS, 1% pen/strep and the indicated compound for 5 days at 37°C. Before starting the incubation, the plate with the tissue slices was inserted in a sealed chamber and flushed for 3 min with O_2_ (3 L/min). After the 5 days, the tissues were collected and fixed for 2 h in 4% PFA under constant agitation, then washed in PBS, dehydrated and embedded in paraffin.

### Flow Cytometry

Single cells from dissociated organoids or from digested tissues were washed in FACS buffer (0.5% BSA, 2 mM EDTA in PBS, pH 7.4). Cells were resuspended in a total of 100 μl of FACS buffer with anti-CD44-APC (1:20, BD Bioscience, clone G515) and incubated for 20 min in the dark at room temperature. Cells were then washed once in FACS buffer before proceeding to ALDEFluor staining, as per manufacturer's indications. Tubes were incubated for 45′ at 37°C. After the incubation, cells were washed in ALDEFluor buffer (AB) and resuspended in 300 μl of AB per tube, supplemented with 5 μg/mL of DAPI and kept on ice until acquisition with a BD LSRII flow cytometer (BD Biosciences).

### Data Analysis

Data was analyzed using Prism GraphPad 8. Flowcytometry data was analyzed using FlowJo v. 10.6.2. All samples acquired by flow cytometry were analyzed with technical gates by the identification of the population of interest in a SSC-A/FSC-A dot plot, followed by a doublets-excluding gate in a FSC-H/FSC-A dot plot and by a viability gate for DAPI exclusion in a DAPI-A/FSC-A dot plot. For samples stained with ALDEFluor, a minimum of 100,000 events was acquired, for other samples a minimum of 30,000 was acquired, experiments were run in biological duplicates. Gating for ALDEFluor-high (ALDH-hi) cells was setup in the DEAB-treated, matched control sample using median and robust standard deviation (rSD) of fluorescence according to the following formula:

ALDH-hi threshold = (FITC_[Median]_ of DAPI-negative cells) + (3^*^FITC_[rSD]_ of DAPI-negative cells)

Ki67 immunohistochemistry was quantified with ImageJ (v1.52p). A macro was developed to semi-automatically segment and quantify nuclei and AEC signal. Staining is reported as fraction of Ki67-positive nuclei over total counted nuclei, quantifying at least 5 fields per condition.

## Results

### Bone Metastasis From Breast and Prostate Cancer Have Distinctive Molecular Signatures

**Figure 1:**
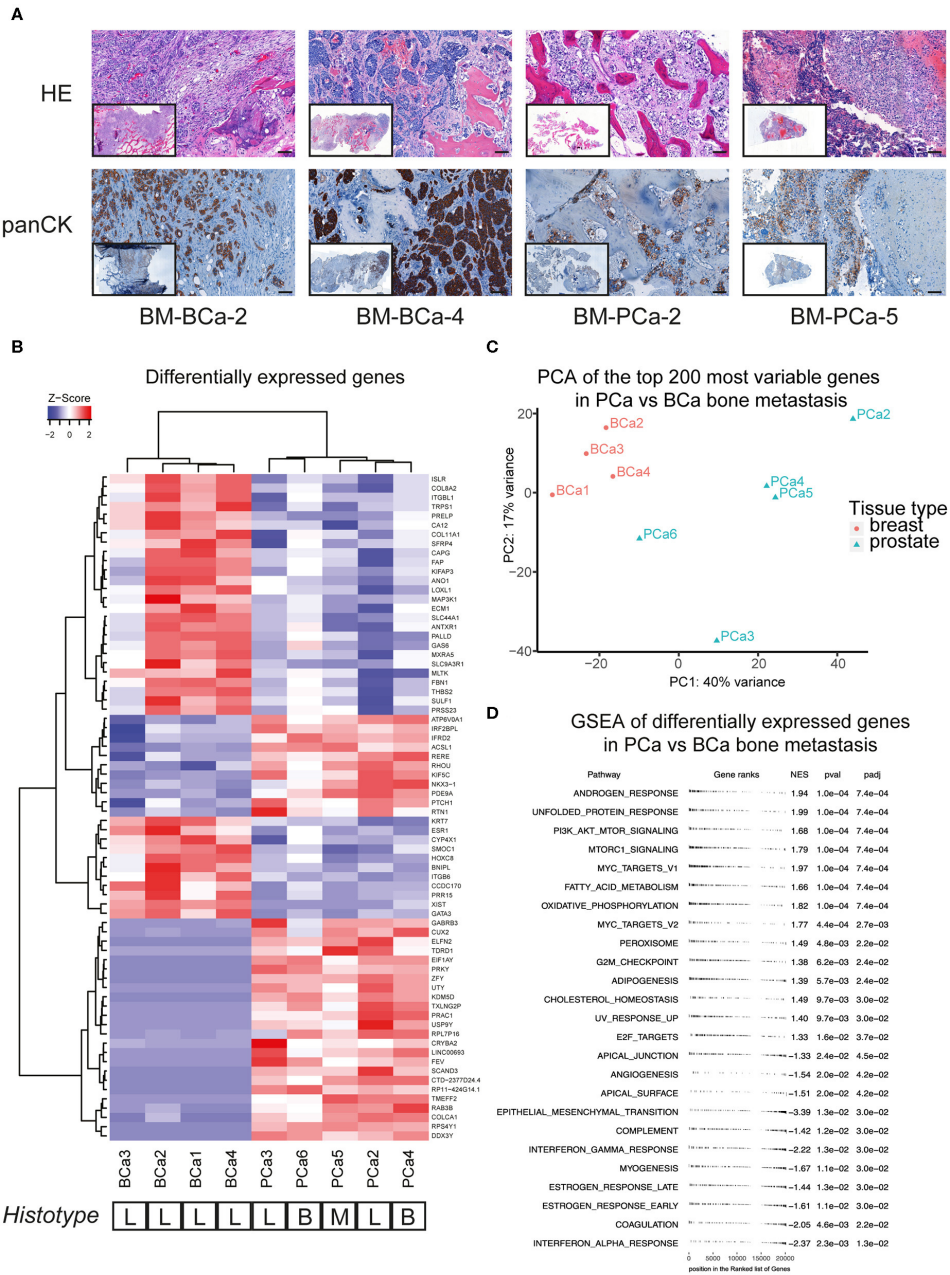
**(A)** Histological sections of breast and prostate bone metastasis. Sections were stained with hematoxylin and eosin (upper row) and for cytokeratin expression (lower row). Whole-section image included in caption. Scale bar 100 μm. **(B)** Analysis of differentially expressed genes of breast and prostate bone metastasis, with unsupervised cluster analysis of both samples and genes. The histological type of bone lesion is reported below the heatmap and is referenced as lytic (L), blastic (B), or mixed (M). **(C)** Principal component analysis (PCA) of the top-200 most differentially expressed genes between the BCa and PCa bone metastasis samples included in **(B)**. While all BCa bone metastasis form a defined cluster, PCa bone metastasis show a more scattered profile that does not recapitulate their histological subtype. **(D)** Geneset enrichment analysis (GSEA) of differentially expressed genes shown in **(B)**. Scores > 0 identify genesets enriched in the prostate bone metastasis group, while scores <0 identify genesets enriched in the breast bone metastasis group. Only significantly enriched genesets are shown.

We investigated the molecular profile of snap-frozen bone metastasis specimens from patients with advanced breast or prostate cancer. For a few samples that were available in sufficient amount, a portion of fresh specimen was fixed and paraffin embedded to perform both a hematoxylin and eosin (HE) histological staining and an immunohistochemistry for cytokeratins (panCK) on cut sections (Figure 1A). The detection of cytokeratin-positive cells as well as the overall poorly organized bone structure in the analyzed sections confirmed the presence of epithelial cells in the bone sample and a pathological, metastasis-induced bone remodeling process. We performed RNASeq analysis on the bone metastasis specimens, the most differentially expressed genes among the included samples are reported (Figure 1B).

The samples formed two subgroups by unsupervised cluster analysis, reflecting the primary cancer of origin. Of note, the bone metastasis samples from prostate cancer did not cluster according to their histotype (lytic, blastic, or mixed lesions), rather by molecular features. The molecular clustering was further investigated by principal component analysis of the top 200 most differentially expressed genes, between the BCa and PCa bone metastasis samples. While all BCa bone metastasis samples formed one cluster, the PCa bone metastasis showed a more scattered distribution, that did not correspond to the histological bone lesion type (Figure 1C). Further pathways analysis on the differentially expressed genes in metastatic PCa highlighted the enrichment of androgen response genes, together with processes linked to lipid metabolism (adipogenesis, cholesterol homeostasis, peroxisome, fatty acid metabolism, Figure 1D). Moreover, PCa bone metastasis showed a specific enrichment for the mTOR pathway, compared to BCa bone metastasis, which showed a specific enrichment for inflammatory processes (interferon response, angiogenesis) and for genes involved in epithelial-to-mesenchymal transition.

### Dual mTORC1/mTORC2 Blockade and ALDH Inhibition Reduce Advanced PCa Organoids Viability *in vitro*

**Figure 2:**
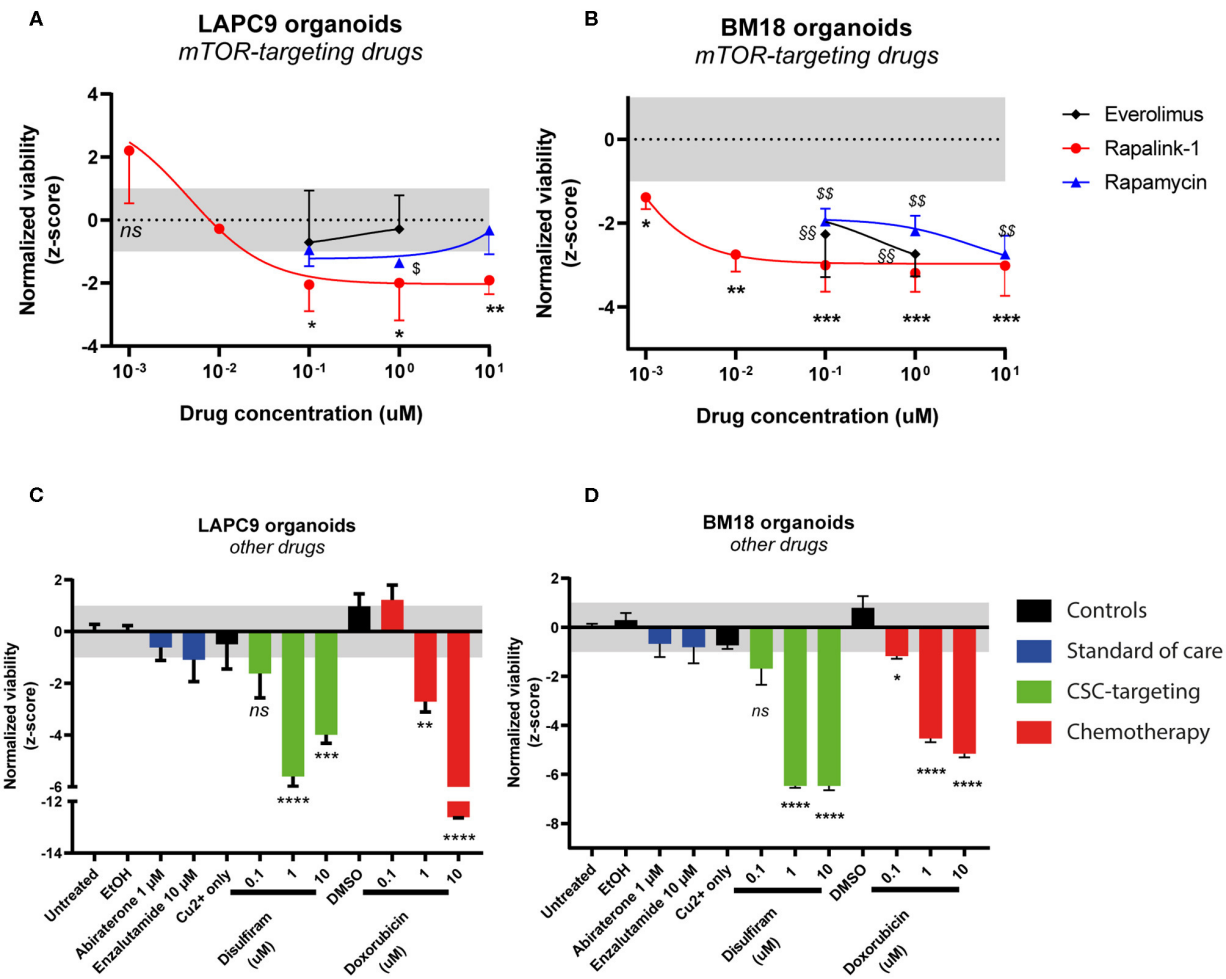
Dose-response curves of LAPC9 **(A)** and BM18 **(B)** PDX organoids to mTOR-targeting drugs rapalink-1 (red circles), rapamycin (blue triangles), and everolimus (black diamonds) after 48 h treatment. Normalized viability values of organoids are plotted against log_10_ drug concentrations; *N* = 2–3. Viability assay of LAPC9 **(C)** and BM18 **(D)** PDX organoids after treatment for 48 h with other, non mTOR-targeting drugs. The grayed area in plots **(A–D)** corresponds to the expected distribution of the reference condition (untreated). Data were analyzed by one-way ANOVA, treated conditions were compared to vehicle (DMSO, EtOH for abiraterone treatment); *N* = 2–3. *p < 0.05; **, §§*p* < 0.01; ***p < 0.001; ****p < 0.0001.

We investigated the effects of mTOR-targeting drugs rapamycin, everolimus and Rapalink-1, a 3^rd^ generation dual mTORC1/2 inhibitor on BM18 and LAPC9 PDX, *in vitro* on organoids. Drug assays on PDX organoids indicated that both LAPC9 and BM18 organoids viability was significantly reduced when treated with Rapalink-1, with a higher IC_50_ in LAPC9 organoids (0.0046 μM) compared to BM18 organoids (0.0003 μM). LAPC9 organoid viability could not be reduced by everolimus at the tested concentrations and could be significantly reduced by rapamycin only at 1 μM, evidencing on the other hand an average viability at 10 μM. BM18 instead showed significant reduction of organoid viability when treated with either everolimus or rapamycin (Figures 2A,B).

We then assessed the effects of the standard of care drugs (abiraterone and enzalutamide) on organoids from both PDX, comparing them to disulfiram and to doxorubicin, this latter used for its efficacy on both PDX models (Figures 2C,D). After 48 h drug exposure, none of the standard of care drugs had significant impact on organoid viability. On the other hand, doxorubicin effectively and dose-dependently reduced viability of both LAPC9 and BM18 organoids. Treatment with disulfiram 1–10 μM, in presence of 1 μM copper gluconate, also significantly impacted organoid viability in both PDX models and had a dose-dependent effect.

### mTORC1/2 Blockade Alters Multiple Metabolic Pathways in Advanced PCa Organoids *in vitro*

**Figure 3:**
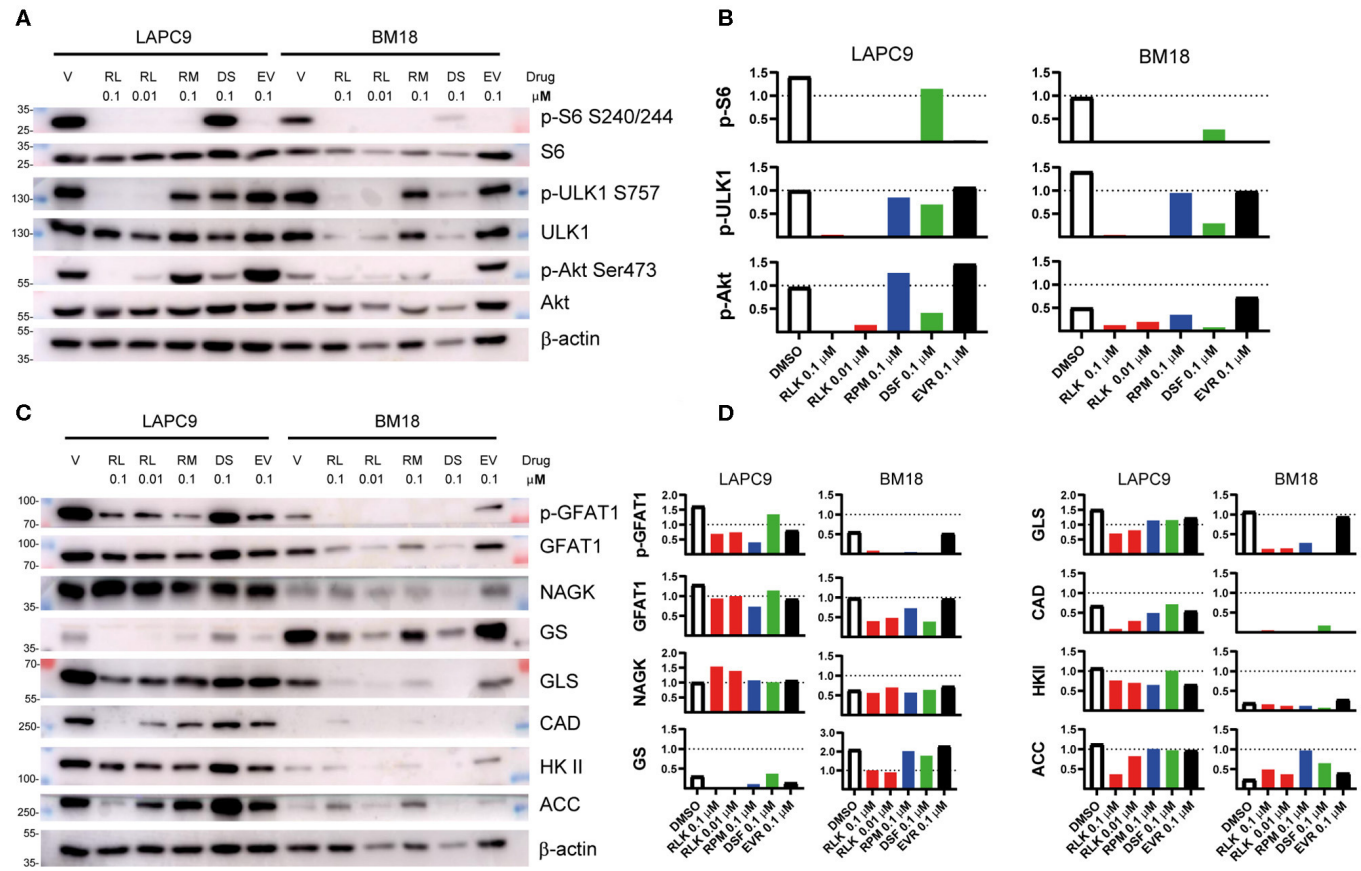
Total lysates of LAPC9 and BM18 organoids treated with rapalink-1 (RL, red bars), rapamycin (RM, blue bars), everolimus (EV, black bars), disulfiram (DS, green bars), or vehicle (V, DMSO, open bars), at the reported concentrations (μM) for 48 h were fractionated by SDS-PAGE followed by Western blotting. Phosphorylated sites or total proteins were detected by immunoblotting using antibodies against the indicated phosphosites or protein. Molecular weight (MW) marker sizes are indicated on the left. Beta-actin was used as loading control. **(A)** The activation status of mTOR was assessed by analyzing the expression of the mTORC1 downstream targets ULK (p-ULK1, S757) and S6 (pS6, S240/244) and of the mTORC2 downstream target Akt (p-Akt, S473). **(B)** Quantification of the phosphosites analyzed in **(A)**. **(C)** The lysates analyzed in **(A)** were further assayed for the activation of the hexosamine biosynthesis pathway (GFAT1; p-GFAT1, S243; NAGK) and of glutamine (GS, GLS), nucleotide (CAD), glucose (HK II), and lipid (ACC) metabolism. **(D)** Quantification of the targets assayed in **(C)**. Glutamine:fructose 6-phosphate Amidotransferase, GFAT1; Glutamine synthetase, GS; Hexokinase II, HK II; acetyl-CoA carboxylase, ACC; Glutaminase, GLS; carbamoyl-phosphate synthetase 2, aspartate transcarbamylase, dihydroorotase, CAD; N-acetyl-Dglucosamine kinase, NAGK.

To determine the mTOR targets that become inhibited by our drug treatment, we performed western blot analyses on LAPC9 and BM18 organoids treated for 48 h with DMSO 0.1%, Rapalink-1 0.1–0.01 μM, rapamycin 0.1 μM, everolimus 0.1 μM, or disulfiram 0.1 μM. In both PDX models, treatment with Rapalink-1 efficiently blocked phosphorylation of the mTORC1 effectors, S6 (Ser240/244) and ULK1 (Ser757). It also abolished Akt phosphorylation (Ser473) in a dose-dependent way (Figures 3A,B). Although rapamycin and everolimus abolished S6 phosphorylation, they had little to no effect on ULK1 phosphorylation in both LAPC9 and BM18. They also did not reduce Akt phosphorylation and in fact everolimus slightly enhanced Akt phosphorylation. In contrast, treatment with disulfiram had little to no effect on phosphorylation of S6 and ULK1, but reduced Akt phosphorylation in both models. Together, these findings demonstrate that Rapalink-1, but not the rapalogues or disulfiram, can effectively block both mTORC1 and mTORC2 signaling in the PDX organoids.

Since mTOR controls metabolism, we investigated how different metabolic enzymes could be affected by our drug treatment. Consistent with the robust inhibition of mTORC2 by RapaLink-1 and previous reports that mTORC2 responds to glutamine catabolites (35), we found that the metabolic enzymes that are linked to glutamine metabolism such as GFAT1, GS, GLS, and CAD were effectively diminished by RapaLink-1 (Figures 3C,D). Furthermore, ACC, a metabolic enzyme involved in lipid metabolism, which is also controlled by both mTORC1 and mTORC2 (36–38), was also reduced by Rapalink-1 but not by rapamycin or everolimus. On the other hand, NAGK, which is involved in the salvage hexosamine biosynthesis and HK II, which is involved in glucose metabolism were not significantly affected by any of the drug treatments. Thus, inhibiting both the mTOR complexes using Rapalink-1 can more effectively block the expression of metabolic enzymes involved in glutamine and lipid metabolism.

### Combined Inhibition of mTORC1/2 Decreases Stem Cell Markers in a CRPC PDX Model and Reduces PCa Bone Metastasis PDO Viability

**Figure 4:**
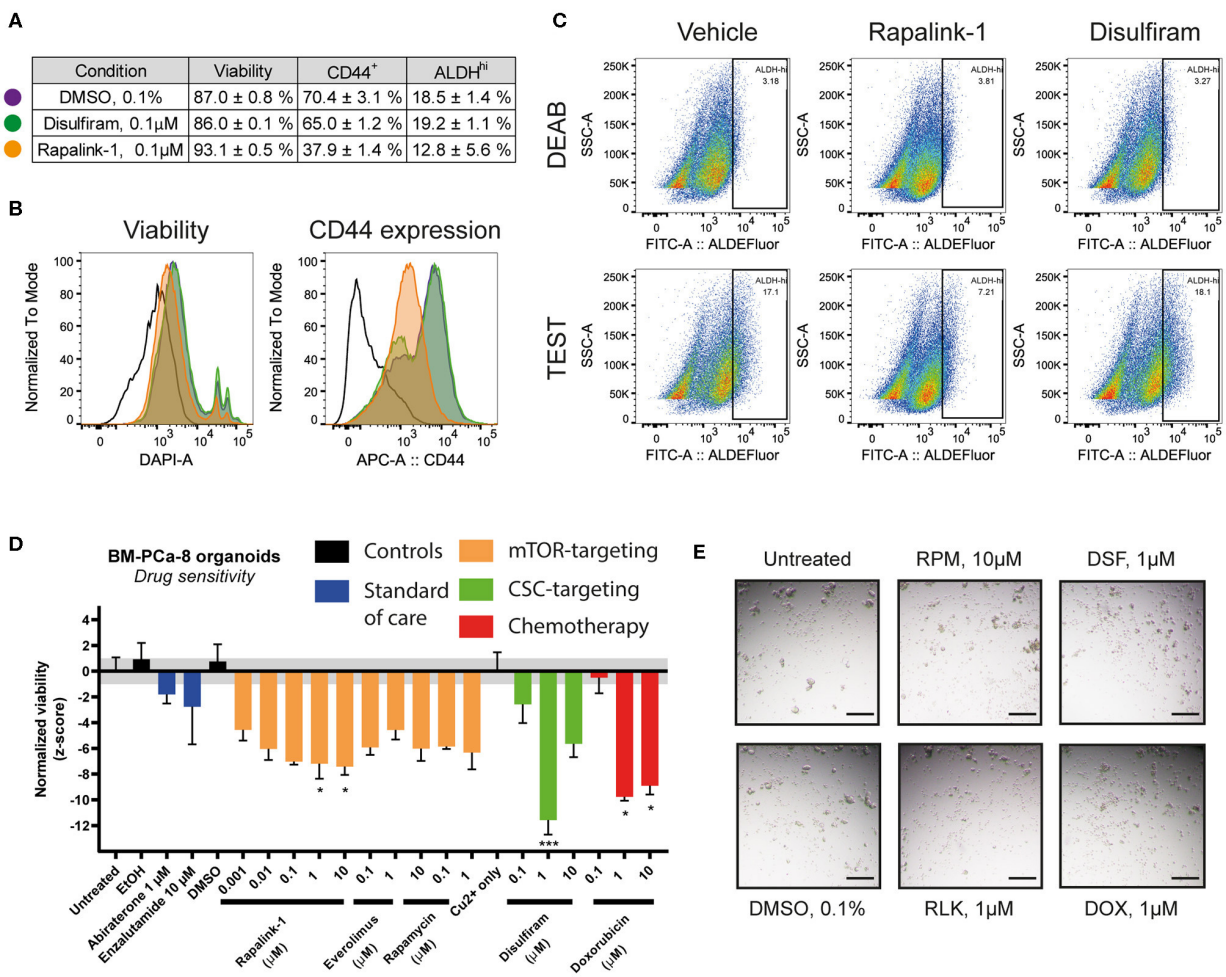
**(A)** Summary table of viability values and of CD44^+^ and ALDH^Hi^ cells in LAPC9 organoids cultures treated with DMSO 0.1% (purple circle), disulfiram 0.1 μM (green circle) or Rapalink-1 0.1 μM (orange circle) for 48 h before analysis via flow cytometry. Data are reported as mean ± SEM, *N* = 2–5. **(B)** Representative ALDEFluor staining dot plots for ALDH^Hi^ determination reported in **(A)**; open histogram, unstained; purple, DMSO; green, disulfiram; orange, Rapalink-1. Rapalink-1 reduced the ALDH^Hi^ population of cells, although not significantly. **(C)** Representative viability and CD44 expression plots of data reported in **(A)**. Treatment of LAPC9 organoids with Rapalink-1 for 48 h significantly increased viability and reduced CD44 expression (*p* < 0.0001 for both analyses). Color code for the histograms is the same as reported in **(A)**. Drug screen assay on prostate bone metastasis organoids from sample BM-PCa-8. Organoids were seeded and allowed to form for 48 h before treating with the reported drugs for 48 h. **(D)** Viability values of organoids across the various tested conditions were normalized and shown. The grayed area corresponds to the expected distribution of the reference condition (untreated). Standard of care drugs (blue) were not effective treatments *in vitro* at the tested concentrations, while among the mTOR-targeting drugs (orange) the highest concentrations of rapalink-1 significantly reduced organoids viability after 48 h of treatment. Treatment with doxorubicin (1, 10 μM, red) as well as with the cancer stem cell-targeting drug disulfiram (1 μM, with 1 μM copper gluconate, green) significantly reduced PCa bone metastasis organoids viability. Data were analyzed by one-way ANOVA, treated conditions were compared to vehicle (DMSO). *N* = 2; *p < 0.05; ***p < 0.001. **(E)** Representative images of BM-PCa-8 organoids after 48 h of treatment with the indicated drugs. Scale bars, 200 μm.

To further investigate the effects of the combined mTORC1/2 inhibition on the cancer stem cell (CSC) subpopulation, we treated LAPC9 organoids for 48 h with sublethal doses of Rapalink-1 (0.1 μM), comparing the effect to treatment with disulfiram (0.1 μM, with copper gluconate 1 μM) or DMSO (0.1%). The treated organoids were then analyzed with flow cytometry for viability, ALDEFluor staining and CD44 expression. A table summarizing the results is reported in Figure 4A.

We found that compared to DMSO, treatment with disulfiram had no impact on the assessed markers, whereas treatment with Rapalink-1 significantly reduced the CD44-positive cell fraction (from 70.4 to 37.9%, *p* < 0.0001) and increased viability (from 87.0 to 93.1%, *p* < 0.0001, Figure 4B). It also decreased the fraction of ALDH^Hi^ cells, although with a higher variability compared to the other markers (from 18.5 to 12.8%, *ns*, Figure 4C). Data from the BM18 PDX were also generated and despite a trend in reduced CD44-positive cell fraction and ALDH^Hi^ cells, comparing the effects of Rapalink-1 and disulfiram to DMSO yielded no significant differences (Supplementary Figure 1)

We functionally tested the effect of Rapalink-1 on CSC by performing a drug assay on PDO from a PCa bone metastasis sample (BM-PCa-8, Figure 4D). We found that treating the organoids with abiraterone or enzalutamide, two standard of care drugs normally used for the treatment of advanced castration-resistant PCa (CRPC), had no significant effect on their viability, supporting a castration-resistant profile for this sample. Doxorubicin, as well as the CSC-targeting drug disulfiram, were both effective to reduce BM-PCa organoids viability. For this latter drug, multiple cytotoxic mechanisms of action were proposed, both dependent and independent on copper that was supplemented in the disulfiram-treated wells (18). Representative pictures of the BM-PCa-8 PDO after 48 h treatment with the reported drugs are shown in Figure 4E.

### Treatment of LAPC9 *in vivo* With Rapalink-1 Delays Tumor Growth

**Figure 5:**
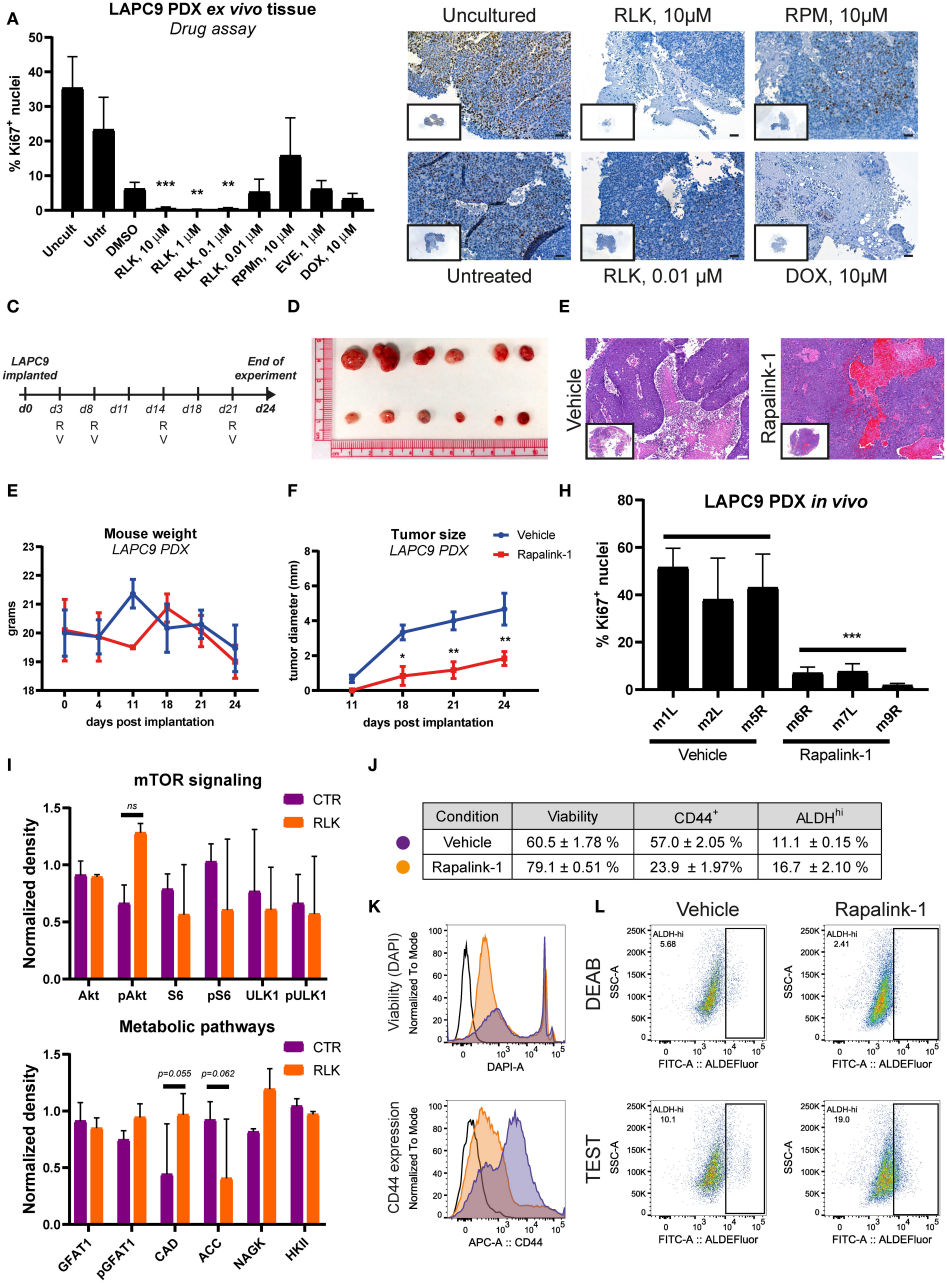
**(A)** Quantification of Ki67-positive nuclei stained on LAPC9 *ex vivo* tissue slices treated with the indicated compounds; a minimum of 5 fields per condition were analyzed. Data are reported as mean ± SD **(B)**. Representative images of Ki67-stained uncultured and untreated tissue, as well as of tissue treated with Rapalink-1 (RLK), rapamycin (RPM), and doxorubicin (DOX) at the reported concentrations are included, with full section enclosed in caption. Scale bar, 50 μm. **(C)** Schematic of the *in vivo* mouse experiment. R and V indicate administration of Rapalink-1 or vehicle, respectively, at the indicated day. **(D)** Picture of LAPC9 tumors explanted from Rapalink-1-treated (bottom) and vehicle-treated groups (top) at the end of experiment. Mice weight curves **(E)** and tumor size measurement **(F)** of bilateral LAPC9 PDX tumors in mice treated with vehicle (blue) or Rapalink-1 (red). For each group, *n* = 3 mice; Data are reported as mean ± SEM. **(G)** Representative HE stainings of LAPC9 tumors from vehicle-treated (top) and Rapalink-treated (bottom) mouse groups. Scale bar, 100 μm. **(H)** Quantification of Ki67-positive nuclei stained on FFPE sections of LAPC9 tissues from mice treated with Rapalink-1 or vehicle; a minimum of 9 fields per sample were analyzed. **(I)** Quantification of western blots of LAPC9 lysates from mice treated with Rapalink-1 or vehicle. Signal from assessed targets was normalized to loading control (beta actin) for each lane **(J)**. Summary table of viability values and of CD44^+^ and ALDH^Hi^ cells in LAPC9 tumors from mice treated with vehicle (purple circles) or Rapalink-1 (orange circles), analyzed by flow cytometry. Data are reported as mean ± SEM, *N* = 2–4. Representative flow cytometry plots of viability and CD44 expression are reported in **(K)**. **(L)** Representative ALDEFluor staining dot plots for ALDH^Hi^ determination reported in **(J)**; open histogram, unstained; orange, Rapalink-1; purple, vehicle. LAPC9 cells from tumors of mice treated with Rapalink-1 showed a non-significant increase of ALDH^Hi^ cells. A population bearing DEAB-resistant ALDH isoforms is detected in LAPC9 tumors of mice treated with vehicle (top, left panel) that is not evident in the tumors of mice treated with Rapalink-1 (top, right panel). *p < 0.05; **p < 0.01; ***p < 0.001.

Before evaluating the effects of Rapalink-1 *in vivo*, we investigated its effects on a near-patient *ex vivo* tissue slices assay on the PDX LAPC9. We compared the effects of Rapalink-1 to those of rapamycin, everolimus and doxorubicin, selected as a positive control. The effect of the drugs on the proliferation marker Ki67 was measured on FFPE sections of the treated *ex vivo* tissue. Treatment with Rapalink-1 at the three highest concentrations tested (10–0.1 μM) significantly reduced LAPC9 proliferation *ex vivo*, in line with the effective inhibition of mTOR signaling and expression of several metabolic enzymes (Figure 5A). Representative images of the *ex vivo* LAPC9 tissues treated with the reported drugs are shown in Figure 5B, an image of the whole section is enclosed. Representative output images of ImageJ macro quantification after processing are reported in Supplementary Figure 2.

We then assessed the effect of Rapalink-1 (1.5 mg/Kg/5–7 days) *in vivo* on LAPC9 PDX model, comparing the treatment to vehicle only, a schematic of treatment schedule is reported (Figure 5C). At the end of the experiment, mice treated with Rapalink-1 had significantly smaller tumors compared to mice treated with vehicle only (Figures 5D,F). Mice treated with Rapalink-1 did not show signs of acute toxicity throughout the experiment and had a weight curve comparable to that of vehicle-treated animals (Figure 5E). Basing on hematoxylin and eosin (HE) staining, LAPC9 tissues collected from the Rapalink-treated mouse group showed a lower fraction of necrotic tissue compared to stainings from the vehicle group (Figure 5G). Analysis of tumors from mice treated with Rapalink-1 showed a significantly lower proliferative activity, as evidenced by Ki67 staining on FFPE tumor sections (Figure 5H, Supplementary Figure 3). Protein analysis of matched tumor lysates showed diminished phosphorylation of S6 and ULK1, indicating inhibition of mTORC1. Interestingly, Akt phosphorylation was enhanced in the Rapalink-treated group, indicating that mTORC2 was active at this time point (Figure 5I, Supplementary Figure 4). Among the metabolic enzymes that we examined, there was an increase of nitrogen metabolizing enzymes CAD and NAGK and a decrease of ACC1, controlling lipid biosynthesis. In order to assess the effect of Rapalink-1 treatment on the CSC subpopulation of LAPC9, tumors from Rapalink-1 and vehicle-treated animals were digested and stained for CD44 expression and with ALDEFluor assay, whereas DAPI was used to measure cell viability within the analyzed samples. Two samples per condition were independently processed and acquired, a table with the results is reported together with representative plots of viability measurement and of CD44 expression (Figure 5J). Tumor cells from mice treated with Rapalink-1 had on average a significantly higher viability compared to tumors from vehicle-treated animals (79.1 ± 0.51% vs. 60.5 ± 1.78% alive cells, respectively, *p* < 0.0001). However, the CD44^+^ compartment in the former samples was markedly and significantly lower (23.9 ± 1.97% vs. 57.0 ± 2.05%, respectively, *p* < 0.0001), indicating a depletion of CD44^+^ cells in the LAPC9 tumors of mice treated with Rapalink-1 (Figure 5K). Unexpectedly, the ALDEFluor assay indicated an enrichment, although not significant, of ALDH^hi^ cells in the Rapalink-treated tumors (16.7 ± 2.10%) compared to the vehicle-treated tumors (11.1 ± 0.15%). Of note, the ALDEFluor assay reveals that treatment of mice with Rapalink-1 induced a metabolic alteration in LAPC9 cancer cells. This was highlighted by the presence of a DEAB-resistant, ALDEFluor-reactive subpopulation of cells clearly detectable in the DEAB-treated samples of mice receiving vehicle (Figure 5L, top left panel). A DEAB-resistant population was not detectable in LAPC9 cells of mice treated with Rapalink-1 (Figure 5L, top right panel).

## Discussion

Despite the intense research on the mechanisms of bone metastasis formation, a consensus molecular classification of bone metastasis is still missing. Metastatic bone lesions can be histologically identified as lytic, blastic or mixed if the effect on the bone tissue is mainly erosive, sclerotic or a co-occurrence of both processes, respectively (1). In this work however, this histological classification did not match the unsupervised cluster analysis nor the PCA at the transcriptomic level, in contrast to the findings of a recent study by Ihle et al. (36). In their study, the authors compared lytic and blastic PCa bone metastasis by GSE analysis, finding the enrichment of different pathways in the lytic vs. blastic lesions. The differences found between the present study and that from Ihle *et al*. may be ascribed to the different sources used in the two settings (FFPE vs. snap-frozen tissue). We demonstrated the cytotoxic effectiveness of Rapalink-1, in comparison to doxorubicin, disulfiram and standard-of-care drugs, on PCa established PDX and near-patient bone metastasis-derived organoids. Standard-of-care drugs abiraterone and enzalutamide could not elicit a significant response in any of the tested conditions. As both the established PDX and the bone metastasis organoids are derived from advanced, bone-metastatic prostate cancer, this result might reflect convergent resistance mechanisms to AR inhibition possibly evolved during tumor progression. This is particularly significative for BM18 organoids, as this model is sensitive to AR inhibition *in vivo* (8, 35). The organoids *in vitro* culture system may enrich for a more AR independent subpopulation, as in the *in vivo* castrated state. As expected however, all organoids responded to the chemotherapeutic drug doxorubicin targeting DNA replication.

While organoids from both PDX were significantly inhibited by disulfiram concentrations above 1 μM, in bone metastasis organoids the significant cytotoxic effect shown at 1 μM was not replicated at the higher concentration of 10 μM. This could be explained by the chemistry of disulfiram, that forms cytotoxic equimolar complexes with Cu^2+^. At 10 μM concentration the amount of uncomplexed disulfiram might have reduced the cytotoxic effect of Cu^2+^-complexed disulfiram. More significantly, the sublethal dose of 0.1 μM disulfiram tested *in vitro* on LAPC9 and BM18 organoids failed to significantly modulate CSC features like CD44 expression or ALDH^Hi^ cell fraction. Overall, these results suggest multiple mechanisms of action of disulfiram, that could be linked to concentration and bioavailability.

Activating mutations in different components of the PI3K/Akt pathway occur in 49% of mCRPC, including mutations of *PTEN* (>40% of cases), and are solidly implicated in PCa progression (37, 38). The modulation of the PI3K/Akt/mTOR pathway during PCa progression also correlates with alterations in the AR pathway and the cross-talk of these two pathways is currently the focus of active research (27, 39, 40). Increased mTOR signaling is associated with lymph node progression and increased lymphangiogenesis in advanced prostate cancer, supporting a link between mTOR activation and metastatic spread of PCa (41). We confirmed the activation of the PI3K/Akt/mTOR pathway in our group of bone metastatic PCa samples. Noteworthy, in all models tested, including the PCa bone metastasis, we found increased sensitivity to Rapalink-1 compared to rapamycin and the rapalog everolimus. Several clinical trials utilizing rapalogs either as monotherapy or combination therapy revealed clinical inefficacy in the treatment of prostate cancer (28) as well as other types of cancer (19). Rapalogs only inhibit a subset of mTORC1 targets and thus have cytostatic rather than cytotoxic effects. Hence, mTOR inhibitors that block mTOR kinase activity have been engineered to more fully inhibit mTOR functions. Since the mTOR kinase domain displays homology to PI3K catalytic domain, dual PI3K/mTOR inhibitors have also been developed for better targeted therapy. However, despite the potent effect of mTOR and PI3K/mTOR inhibitors in cellular models, they have less durable effect *in vivo*, thus necessitating increased dose leading to toxicity (29). Rapalink-1 was developed to combine the durable effect of rapalogs (owing to binding with FKBP12) and robust inhibition of both mTORC1 and mTORC2 (31). The effect of Rapalink-1 in abolishing phosphorylation of mTORC1 (S6, ULK1) and mTORC2 (Akt) effectors was dose-proportionate and coincided with the robust overall decrease in cell viability of the PDX organoids. This was accompanied by significant reduction of expression of metabolic enzymes that have been linked to mTOR signaling, in particular glutamine-requiring pathways and lipid metabolism (19, 20). It is notable that we also found enrichment of genes relating to lipid metabolism and Myc. The latter is involved in increased glutamine metabolism in a number of cancers (42). Hence, it is possible that growth of the bone metastatic PCa organoids is highly dependent on mTOR-mediated glutamine- and/or lipid metabolism, making them particularly susceptible to combined mTORC1/2 inhibition.

The analysis of LAPC9 tumor lysates from mice treated with Rapalink-1 indicates residual mTORC2 activity as well as fewer metabolic alterations compared to Rapalink-1-treated organoids. Despite this divergence, treatment of mice with Rapalink-1 every 6 days was sufficient to significantly reduce tumor growth, as assessed by both tumor size and Ki67 staining on lysates-matched tumor sections. Of note, the analyzed tumor lysates from the Rapalink-1-treated mice group showed that the effects on mTOR activation and lipid metabolism regulation were heterogeneous (Supplementary Figure 4). This could be explained by differences in Rapalink-1 bioavailability among the particular mice, owing to factors like varying tumor size, structure or vascularization, as well as by the onset of compensatory mechanisms in tumors from treated mice. Moreover, both LAPC9 PDX and organoids treated with Rapalink-1 showed a significant decrease of CD44^+^ cells, indicating not only a direct cytotoxic effect of the treatment, but also the alteration of PCa subpopulation homeostasis. In line with this observation, *in vivo* Rapalink-1 treatment altered the expression of aldehyde dehydrogenases (ALDH) in the surviving cells, as evidenced by the ALDEFluor assay. In the assay, the large-spectrum ALDH inhibitor DEAB is provided together with a fluorogenic substrate detecting multiple ALDH isoforms. However, the DEAB does not inhibit all isoforms of ALDH (17), an effect that was evident in the reported results. Recently, Vaddi et al. published a study linking functional CSC traits of multiple PCa cell lines to an enriched PI3K/Akt/mTOR pathway both at the RNA and at the protein levels (43). Of note, pharmacological inhibition of the PI3K/Akt pathway was associated with a reduction of the CSC population *in vitro*, in line with previous reports from Dubrovska et al. (44, 45). The effect of Rapalink-1 on different LAPC9 subpopulations could also explain the small but significant increase of viability detected both *in vitro* at sublethal doses of Rapalink-1 and *in vivo*. In both cases, the dose of Rapalink-1 used could have had a direct cytotoxic effect on the more mTOR-addicted subpopulations, selecting or inducing a subset of CD44-low, metabolically slow cells.

Compared to breast cancer, PCa bone metastasis were also enriched for pathways involved in oxidative phosphorylation and lipid metabolism (fatty acid metabolism, peroxisome, adipogenesis, cholesterol homeostasis), a finding in line with an increase in lipid metabolism in more advanced PCa stages (46) and supporting the clinical relevance of targeting this metabolic branch to prevent the development of androgen-resistance (47). An altered lipid metabolism has been linked to CSC for multiple cancer types (48). Given the substrate preferences of the different ALDH isoforms (49), it would be interesting to determine if upregulation of the mTOR pathway induced metabolic rewiring in PCa cells, or metabolic diversification of subpopulations within the tumor. Further experiments are required to support this hypothesis.

In conclusion, we provided a molecular analysis of a group of breast and prostate cancer bone metastasis and showed the translational applicability of an organoid-based drug screen on patient-derived bone metastatic tissue. We demonstrated the effectiveness of the dual mTORC1-2 inhibitor Rapalink-1 in reducing PCa tumor growth, an effect that was associated with the depletion of CD44^+^ cells in a PDX model of advanced, bone metastatic PCa.

## Data Availability Statement

The datasets presented in this study can be found in online repositories. The names of the repository/repositories and accession number(s) can be found below: European Genome Archive (https://ega-archive.org/ - Project ID: EGAS00001004431).

## Ethics Statement

Ethical review and approval was not required for the study on human participants in accordance with the local legislation and institutional requirements.

## Author Contributions

FL collected and analyzed most of the data, contributed in designing the experimental aspects. SK, MDM, and FL carried over the animal experiments and generated the data for PDX transcriptomic. IK generated the histological data. MDF analyzed the transcriptomic data. NP and EJ generated, analyzed, and interpreted proteomic data. PK and LB provided the clinical data and specimens. FL, MK, and EJ wrote the manuscript and designed figures. RP provided critical revision of the article. MK and GT supervised the project. MK conceived and designed the study. All authors contributed to manuscript revision, read and approved the submitted version.

## Conflict of Interest

The authors declare that the research was conducted in the absence of any commercial or financial relationships that could be construed as a potential conflict of interest.

## References

[1] Macedo F Ladeira K Pinho F Saraiva N Bonito N Pinto L. Bone metastases: an overview. Oncol Rev. (2017) 11:321. 10.4081/oncol.2017.321 28584570 PMC5444408

[2] Croucher PI McDonald MM Martin TJ. Bone metastasis: the importance of the neighbourhood. Nat Rev Cancer. (2016) 16:373–86. 10.1038/nrc.2016.44 27220481

[3] Weilbaecher KN Guise TA McCauley LK. Cancer to bone: a fatal attraction. Nat Rev Cancer. (2011) 11:411–25. 10.1038/nrc3055 21593787 PMC3666847

[4] Mehra R Kumar-Sinha C Shankar S Lonigro RJ Jing X Philips NE. Characterization of bone metastases from rapid autopsies of prostate cancer patients. Clin Cancer Res. (2011) 17:3924–32. 10.1158/1078-0432.CCR-10-3120 21555375 PMC3117947

[5] Van Allen EM Foye A Wagle N Kim W Carter SL McKenna A. Successful whole-exome sequencing from a prostate cancer bone metastasis biopsy. Prostate Cancer Prostatic Dis. (2014) 17:23–7. 10.1038/pcan.2013.37 24366412 PMC4364998

[6] Roubaud G Liaw BC Oh WK Mulholland DJ. Strategies to avoid treatment-induced lineage crisis in advanced prostate cancer. Nat Rev Clin Oncol. (2017) 14:269–83. 10.1038/nrclinonc.2016.181 27874061 PMC5567685

[7] Brennen WN Isaacs JT. The what, when, and why of human prostate cancer xenografts. Prostate. (2018) 78:646–54. 10.1002/pros.23510 29575112

[8] McCulloch DR Opeskin K Thompson EW Williams ED. BM18: A novel androgen-dependent human prostate cancer xenograft model derived from a bone metastasis. Prostate. (2005) 65:35–43. 10.1002/pros.20255 15800936

[9] Craft N Chhor C Tran C Belldegrun A DeKernion J Witte ON. Evidence for clonal outgrowth of androgen-independent prostate cancer cells from androgen-dependent tumors through a two-step process. Cancer Res. (1999) 59:5030–6. 10519419

[10] Pece S Tosoni D Confalonieri S Mazzarol G Vecchi M Ronzoni S. Biological and molecular heterogeneity of breast cancers correlates with their cancer stem cell content. Cell. (2010) 140:62–73. 10.1016/j.cell.2009.12.007 20074520

[11] Marcucci F Rumio C Lefoulon F. Anti-cancer stem-like cell compounds in clinical development - an overview and critical appraisal. Front Oncol. (2016) 6:115. 10.3389/fonc.2016.00115 27242955 PMC4861739

[12] Jagust P De Luxán-Delgado B Parejo-Alonso B Sancho P. Metabolism-based therapeutic strategies targeting cancer stem cells. Front Pharmacol. (2019) 10:203. 10.3389/fphar.2019.00203 30967773 PMC6438930

[13] Sharpe B Beresford M Bowen R Mitchard J Chalmers AD. Searching for prostate cancer stem cells: markers and methods. Stem Cell Rev Rep. (2013) 9:721–30. 10.1007/s12015-013-9453-4 23775699

[14] Qin J Liu X Laffin B Chen X Choy G Jeter CR. The PSA-/lo prostate cancer cell population harbors self-renewing long-term tumor-propagating cells that resist castration. Cell Stem Cell. (2012) 10:556–69. 10.1016/j.stem.2012.03.009 22560078 PMC3348510

[15] Magnen C Le Bubendorf L Rentsch CA Mengus C Gsponer J Zellweger T. Characterization and clinical relevance of ALDH bright populations in prostate cancer. Clin Cancer Res. (2013) 19:5361–71. 10.1158/1078-0432.CCR-12-2857 23969936

[16] Yan J De Melo J Cutz JC Aziz T Tang D. Aldehyde dehydrogenase 3A1 associates with prostate tumorigenesis. Br J Cancer. (2014) 110:2593–603. 10.1038/bjc.2014.201 24762960 PMC4021532

[17] Zhou L Sheng D Wang D Ma W Deng Q Deng L. Identification of cancer-type specific expression patterns for active aldehyde dehydrogenase (ALDH) isoforms in ALDEFLUOR assay. Cell Biol Toxicol. (2019) 35:161–77. 10.1007/s10565-018-9444-y 30220009 PMC6424948

[18] Triscott J Rose Pambid M Dunn SE. Concise review: bullseye: targeting cancer stem cells to improve the treatment of gliomas by repurposing disulfiram. Stem Cells. (2015) 33:1042–6. 10.1002/stem.1956 25588723

[19] Magaway C Kim E Jacinto E. Targeting mTOR and metabolism in Cancer: lessons and innovations. Cells. (2019) 8:1584. 10.3390/cells8121584 31817676 PMC6952948

[20] Mossmann D Park S Hall MN. mTOR signalling and cellular metabolism are mutual determinants in cancer. Nat Rev Cancer. (2018) 18:744–57. 10.1038/s41568-018-0074-8 30425336

[21] Janku F Yap TA Meric-Bernstam F. Targeting the PI3K pathway in cancer: are we making headway? Nat Rev Clin Oncol. (2018) 15:273–91. 10.1038/nrclinonc.2018.28 29508857

[22] Liu P Cheng H Roberts TM Zhao JJ Targeting the phosphoinositide 3-kinase pathway in cancer. Nat Rev Drug Discov. (2009) 8:627–44. 10.1038/nrd2926 19644473 PMC3142564

[23] Stambolic V Tsao M-S Macpherson D Suzuki A Chapman WB Mak TW. High incidence of breast and endometrial neoplasia resembling human cowden syndrome in pten+/– Mice. Cancer Res. (2000) 60:3605–11. Available online at: https://cancerres.aacrjournals.org/content/60/13/3605 10910075

[24] Carver BS Chapinski C Wongvipat J Hieronymus H Chen YY Chandarlapaty S. Reciprocal feedback regulation of PI3K and androgen receptor signaling in pten-deficient prostate cancer. Cancer Cell. (2011) 19:575–86. 10.1016/j.ccr.2011.04.008 21575859 PMC3142785

[25] Mulholland DJ Tran LM Li Y Cai H Morim A Wang S. Cell autonomous role of PTEN in regulating castration-resistant prostate cancer growth. Cancer Cell. (2011) 19:792–804. 10.1016/j.ccr.2011.05.006 21620777 PMC3157296

[26] Wang X Julio MKD Economides KD Walker D Yu H Halili MVV. A luminal epithelial stem cell that is a cell of origin for prostate cancer. Nature. (2009) 461:495–500. 10.1038/nature08361 19741607 PMC2800362

[27] Audet-Walsh É Dufour CR Yee T Zouanat FZ Yan M Kalloghlian G. Nuclear mTOR acts as a transcriptional integrator of the androgen signaling pathway in prostate cancer. Genes Dev. (2017) 31:1228–42. 10.1101/gad.299958.117 28724614 PMC5558925

[28] Statz CM Patterson SE Mockus SM. mTOR inhibitors in castration-resistant prostate cancer: a systematic review. Targeted Oncol. (2017) 12:47–59. 10.1007/s11523-016-0453-6 27503005

[29] Wei XX Hsieh AC Kim W Friedlander T Lin AM Louttit M. A phase I study of abiraterone acetate combined with BEZ235, a dual PI3K/mTOR inhibitor, in metastatic castration resistant prostate cancer. Oncologist. (2017) 22:503–e43. 10.1634/theoncologist.2016-0432 28314838 PMC5423513

[30] Graham L Banda K Torres A Carver BS Chen Y Pisano K. A phase II study of the dual mTOR inhibitor MLN0128 in patients with metastatic castration resistant prostate cancer. Invest New Drugs. (2018) 36:458–67. 10.1007/s10637-018-0578-9 29508246 PMC6050986

[31] Rodrik-Outmezguine VS Okaniwa M Yao Z Novotny CJ McWhirter C Banaji A. Overcoming mTOR resistance mutations with a new-generation mTOR inhibitor. Nature. (2016) 534:272–6. 10.1038/nature17963 27279227 PMC4902179

[32] Beltran H Eng K Mosquera JM Sigaras A Romanel A Rennert H. Whole-exome sequencing of metastatic cancer and biomarkers of treatment response. JAMA Oncol. (2015) 1:466–74. 10.1001/jamaoncol.2015.1313 26181256 PMC4505739

[33] Dobin A Davis CA Schlesinger F Drenkow J Zaleski C Jha S. STAR: ultrafast universal RNA-seq aligner. Bioinformatics. (2013) 29:15–21. 10.1093/bioinformatics/bts635 23104886 PMC3530905

[34] Nikolayeva O Robinson MD. edgeR for differential RNA-seq and ChIP-seq analysis: an application to stem cell biology. Methods Mol Biol. (2014) 1150:45–79. 10.1007/978-1-4939-0512-6_3 24743990

[35] Karkampouna S La Manna F De Filippo MR Kiener M De Menna M Zoni E Patient-derived xenografts and organoids model therapy response in prostate cancer. bioRxiv [Pre-print]. (2020). 10.1101/2020.03.17.994350 PMC7892572 33602919

[36] Ihle CL Provera MD Straign DM Smith EE Edgerton SM Van Bokhoven A. Distinct tumor microenvironments of lytic and blastic bone metastases in prostate cancer patients. J Immunother Cancer. (2019) 7:293. 10.1186/s40425-019-0753-3 31703602 PMC6839115

[37] Taylor BS Schultz N Hieronymus H Gopalan A Xiao Y Carver BS. Integrative genomic profiling of human prostate cancer. Cancer Cell. (2010) 18:11–22. 10.1016/j.ccr.2010.05.026 20579941 PMC3198787

[38] Robinson D Van Allen EM Wu YM Schultz N Lonigro RJ Mosquera JM. Integrative clinical genomics of advanced prostate cancer. Cell. (2015) 161:1215–28. 10.1016/j.cell.2015.05.001 26000489 PMC4484602

[39] Bitting RL Armstrong AJ. Targeting the PI3K/Akt/mTOR pathway in castration-resistant prostate cancer. Endocr Relat Cancer. (2013) 20:R83–99. 10.1530/ERC-12-0394 23456430

[40] Blattner M Liu D Robinson BD Huang D Poliakov A Gao D. SPOP mutation drives prostate tumorigenesis *in vivo* through coordinate regulation of PI3K/mTOR and AR signaling. Cancer Cell. (2017) 31:436–51. 10.1016/j.ccell.2017.02.004 28292441 PMC5384998

[41] Lilis I Giopanou I Papadaki H Gyftopoulos K. The expression of p-mTOR and COUP-TFII correlates with increased lymphangiogenesis and lymph node metastasis in prostate adenocarcinoma. Urol Oncol Semin Orig Investig. (2018) 36:311.e27–35. 10.1016/j.urolonc.2018.02.007 29544697

[42] Deberardinis RJ Cheng T. Q's next: the diverse functions of glutamine in metabolism, cell biology and cancer. Oncogene. (2010) 29:313–24. 10.1038/onc.2009.358 19881548 PMC2809806

[43] Vaddi PK Stamnes MA Cao H Chen S. Elimination of SOX2/OCT4-associated prostate cancer stem cells blocks tumor development and enhances therapeutic response. Cancers. (2019) 11:1331. 10.3390/cancers11091331 31500347 PMC6769476

[44] Dubrovska A Kim S Salamone RJ Walker JR Maira SM García-Echeverría C. The role of PTEN/Akt/PI3K signaling in the maintenance and viability of prostate cancer stem-like cell populations. Proc Natl Acad Sci USA. (2009) 106:268–73. 10.1073/pnas.0810956106 19116269 PMC2629188

[45] Dubrovska A Elliott J Salamone RJ Kim S Aimone LJ Walker JR. Combination therapy targeting both tumor-initiating and differentiated cell populations in prostate carcinoma. Clin Cancer Res. (2010) 16:5692–702. 10.1158/1078-0432.CCR-10-1601 21138868

[46] Zadra G Photopoulos C Loda M. The fat side of prostate cancer. Biochim Biophys Acta. (2013) 1831:1518–32. 10.1016/j.bbalip.2013.03.010 23562839 PMC3766375

[47] Zadra G Ribeiro CF Chetta P Ho Y Cacciatore S Gao X. Inhibition of *de novo* lipogenesis targets androgen receptor signaling in castration-resistant prostate cancer. Proc Natl Acad Sci USA. (2019) 116:631–40. 10.1073/pnas.1808834116 30578319 PMC6329966

[48] Visweswaran M Arfuso F Warrier S Dharmarajan A. Concise review: aberrant lipid metabolism as an emerging therapeutic strategy to target cancer stem cells. Stem Cells. (2019) 38:6–14. 10.1002/stem.3101 31648395

[49] Koppaka V Thompson DC Chen Y Ellermann M Nicolaou KC Juvonen RO. Aldehyde dehydrogenase inhibitors: a comprehensive review of the pharmacology, mechanism of action, substrate specificity, and clinical application. Pharmacol Rev. (2012) 64:520–39. 10.1124/pr.111.005538 22544865 PMC3400832

