# Corrigendum: Dual-mTOR Inhibitor Rapalink-1 Reduces Prostate Cancer Patient-Derived Xenograft Growth and Alters Tumor Heterogeneity

**DOI:** 10.3389/fonc.2021.650623

**Published:** 2021-02-03

**Authors:** Federico La Manna, Marta De Menna, Nikhil Patel, Sofia Karkampouna, Maria Rosaria De Filippo, Irena Klima, Peter Kloen, Lijkele Beimers, George N. Thalmann, Rob C. M. Pelger, Estela Jacinto, Marianna Kruithof-de Julio

**Affiliations:** ^1^ Department for BioMedical Research, Urology Research Laboratory, University of Bern, Bern, Switzerland; ^2^ Department of Urology, Leiden University Medical Center, Leiden, Netherlands; ^3^ Department of Biochemistry and Molecular Biology, Robert Wood Johnson Medical School, Rutgers, The State University of New Jersey, Piscataway, NJ, United States; ^4^ Institute of Pathology and Medical Genetics, University Hospital Basel, University of Basel, Basel, Switzerland; ^5^ Department of Orthopedic Trauma Surgery, Academic Medical Center, Amsterdam, Netherlands; ^6^ Department of Orthopedic Surgery, MC Slotervaart, Amsterdam, Netherlands; ^7^ Department of Urology, Inselspital, Bern University Hospital, Bern, Switzerland

**Keywords:** bone metastasis, PDX, mTOR, disulfiram, prostate cancer, ALDH

An author name was incorrectly spelled as “**Maria De Filippo”**. The correct spelling is “**Maria Rosaria De Filippo”**.

In the published article, there was also an error in affiliation 1. Instead of “Department of BioMedical Research, University of Bern, Bern, Switzerland”, it should be “Department for BioMedical Research, Urology Research Laboratory, University of Bern, Bern, Switzerland”.

There was also an error in the text. The concentration and administration schedule of Rapalink-1 reported for the *in vivo* experiment was not correct. The error appeared both in the “Materials and Methods” and in the “Results” sections, where it is incorrectly reported as “1.5 mg/g [ … ] every 5 days” and “1.5 mg/g/6 days”, respectively. Figure 5C in the original article reported the correct administration schedule.

A correction has been made to the “Materials and Methods” section, “Animals Maintenance and *in vivo* Experiment” sub-section:

“Group 1 received 3.5 μl/g of vehicle (20% DMSO, 40% PEG-300 and 40% PBS) i.p. once a week while group 2 received Rapalink-1 (1.5 mg/Kg) resuspended in vehicle, i.p. every 5-7 days.”

A correction of the same error has been made to the “Results” section, “Treatment of LAPC9 *in vivo* With Rapalink-1 Delays Tumor Growth” sub-section, paragraph 2:

“We then assessed the effect of Rapalink-1 (1.5 mg/Kg/5-7 days) in vivo on LAPC9 PDX model, comparing the treatment to vehicle only, a schematic of treatment schedule is reported (**Figure 5C**).”

The authors apologize for these errors and state that this does not change the scientific conclusions of the article in any way. The original article has been updated.

