# Supplementary material for: Dual-mTOR Inhibitor Rapalink-1 Reduces Prostate Cancer Patient-Derived Xenograft Growth and Alters Tumor Heterogeneity
Source: Front Oncol. 2020 Jun 23;10:1012. doi: 10.3389/fonc.2020.01012 (PMC7324765; doi:10.3389/fonc.2020.01012)

Supplementary Figure 1

A

Condition	Viability	$CD44^+$	$ALDH^{hi}$
DMSO, 0.1%	68.2 ± 9.5 %	6.2 ± 3.4 %	2.2 ± 1.7 %
Disulfiram, 0.1μM	68.9 ± 9.9 %	5.5 ± 3.0 %	4.4 ± 4.7 %
Rapalink-1, 0.1μM	65.9 ± 12.2 %	4.1 ± 0.6 %	1.4 ± 0.5 %

B

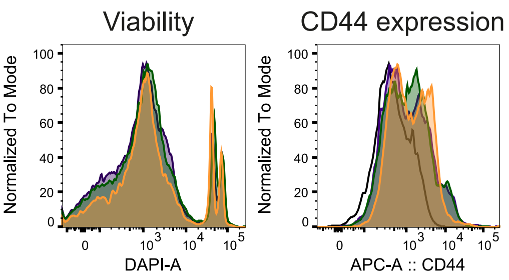

C

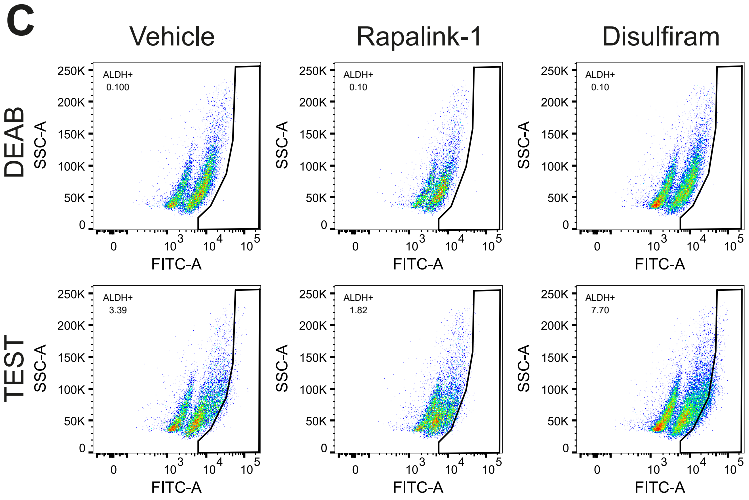

Supplementary Figure 2

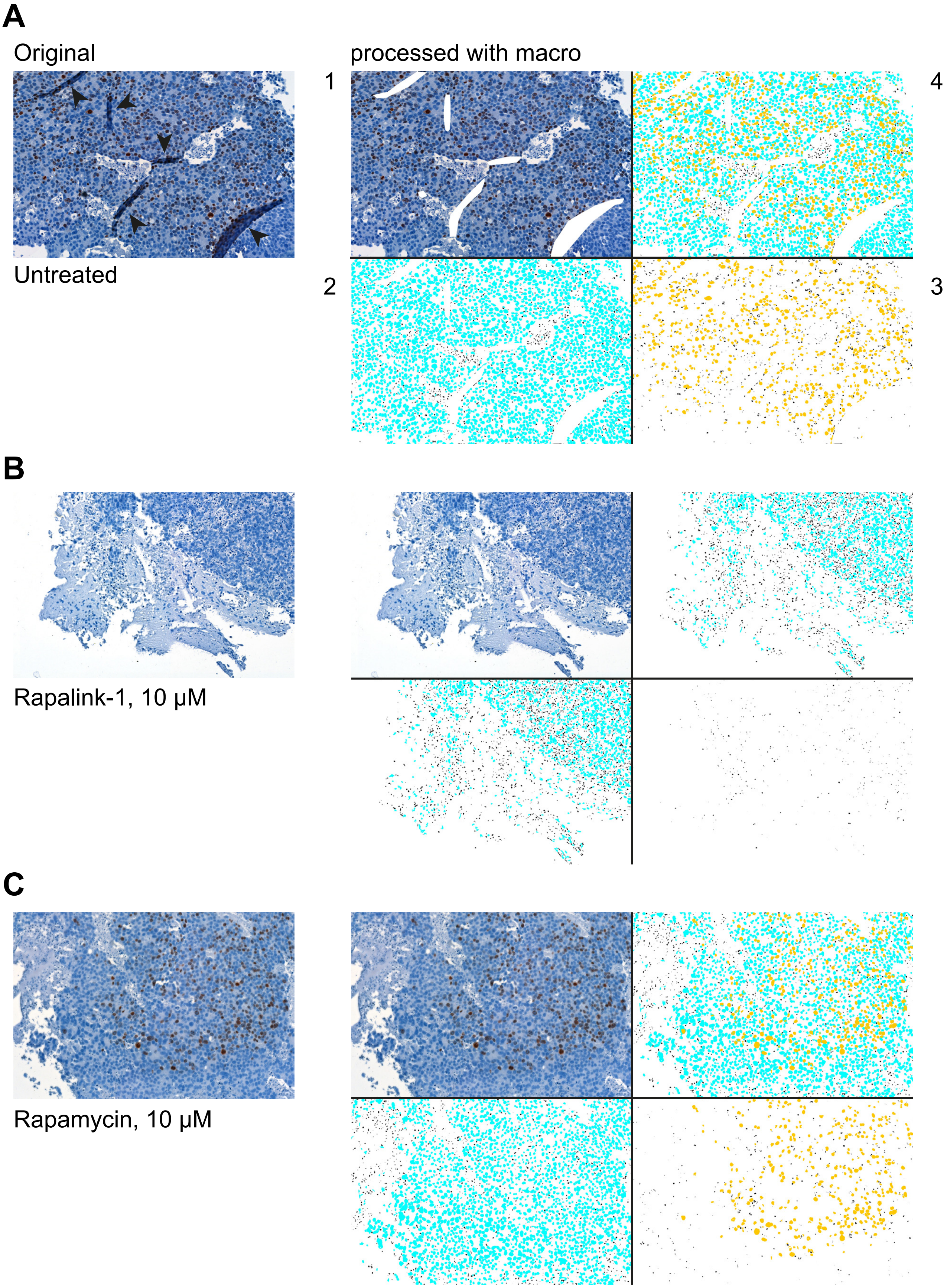

Supplementary Figure 3

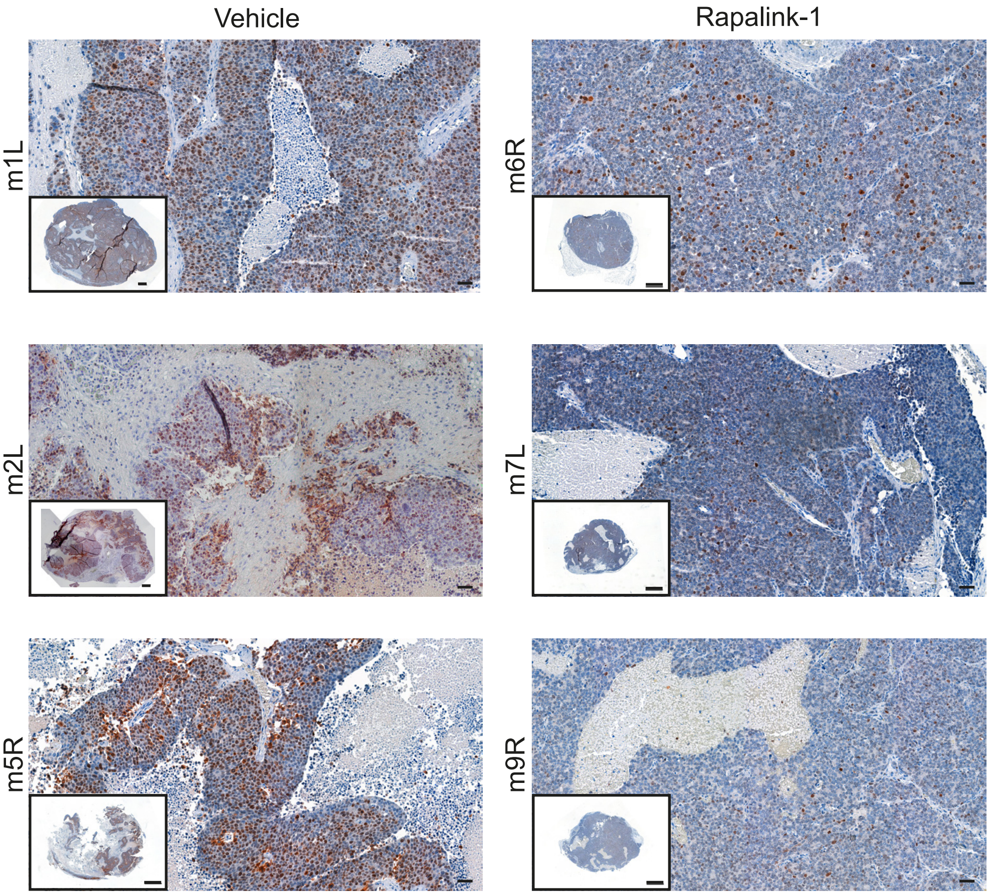

Supplementary Figure 4

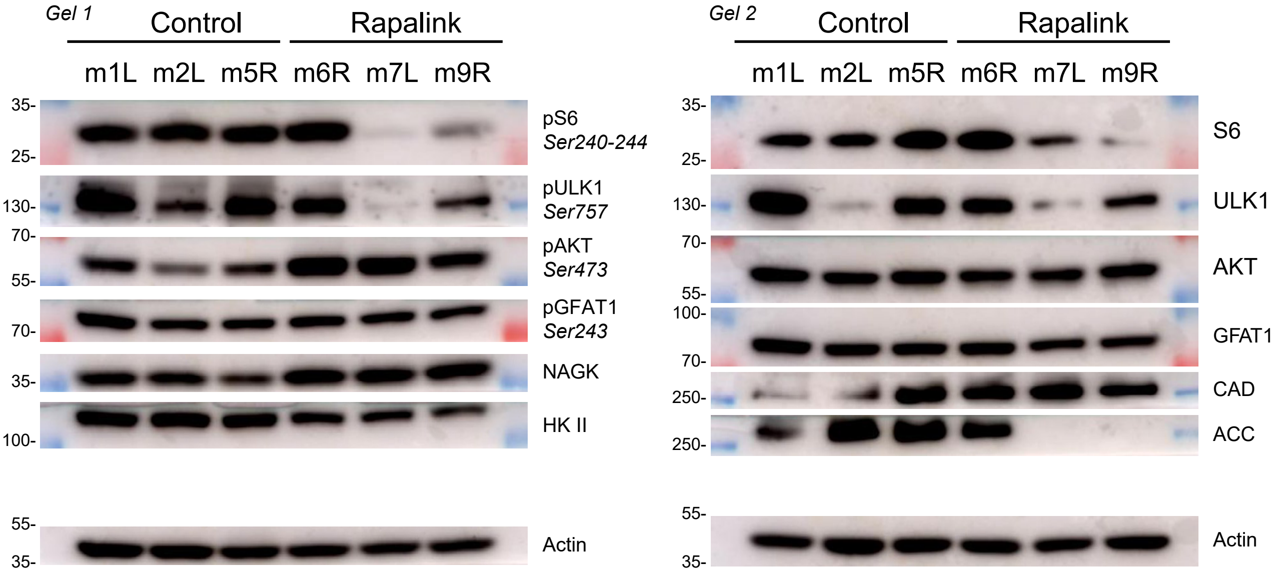

Supplement: Supplementary file 2 [file Data_Sheet_1.pdf]
