# Supplementary material for: Dual-mTOR Inhibitor Rapalink-1 Reduces Prostate Cancer Patient-Derived Xenograft Growth and Alters Tumor Heterogeneity
Source: Front Oncol. 2020 Jun 23;10:1012. doi: 10.3389/fonc.2020.01012 (PMC7324765; doi:10.3389/fonc.2020.01012)
Supplement: Supplementary file 3 [file Data_Sheet_2.docx]

**Supplementary Figures legend**

Supp. Fig. 1

**A**. Summary table of viability values and of CD44^+^ and ALDH^Hi^ cells in BM18 organoids treated with DMSO 0.1% (purple), disulfiram 0.1 µM (green) or Rapalink-1 0.1 µM (orange) for 48h before analysis via flow cytometry. Data are reported as mean ± SD, N=2. **B**. Representative viability and CD44 expression plots of data reported in (**A**). Treatment of BM18 organoids with Rapalink-1 for 48h showed a reduction of CD44 expression, while viability was unaffected. Open histogram, unstained; purple, DMSO; green, disulfiram; orange, Rapalink-1. **C.** Dot plots of ALDEFluor staining on BM18 organoids treated as described in (**A**). A marginal reduction of the ALDH^Hi^ subpopulation is visible in the BM18 organoids treated with Rapalink-1, compared to DMSO and disulfiram (lower panels).

Supp. Fig. 2

Representative images of LAPC9 Ki67 staining from *ex vivo* tissue, untreated (**A**) or after treatment with Rapalink-1 10 µM (**B**) or Rapamycin 10 µM (**C**) and processed with semi-automated ImageJ macro for Ki67 quantification. On the left of each panel the original images are shown, black arrowheads indicate areas with artifacts or dirt potentially interfering with quantification. On the right a collage of output images obtained during processing is reported. Sector 1, original image after areas with artifacts or dirt were manually cropped out. Sector 2, quantified nuclei, shown in cyan. Sector 3, Ki67-positive nuclei, shown in orange. In both sectors 2 and 3, black areas represent items automatically excluded from quantification. Sector 4, overlay of images shown in sectors 2 and 3.

Supp. Fig. 3

Representative images of LAPC9 Ki67 staining from mice treated with Rapalink-1 (right column) or vehicle (left column) *in vivo*. Full-section images are enclosed in bottom left corner of each image. Quantification of Ki67 staining is shown in Fig. 5. Scalebar of close-in images, 50 µm. Scalebar of full-section, 500 µm.

Supp. Fig. 4

Western blots of LAPC9 lysates from mice treated with Rapalink-1 or vehicle *in vivo*. The targets assayed are the same as in Fig. 3.
